# Alteration of gut microbiota affects expression of adiponectin and resistin through modifying DNA methylation in high-fat diet-induced obese mice

**DOI:** 10.1186/s12263-020-00671-3

**Published:** 2020-06-26

**Authors:** Hongyang Yao, Chaonan Fan, Yuanyuan Lu, Xiuqin Fan, Lulu Xia, Ping Li, Rui Wang, Tiantian Tang, Yuanyuan Wang, Kemin Qi

**Affiliations:** 1 Laboratory of Nutrition and Development, Beijing Pediatric Research Institute, Beijing Children’s Hospital, Capital Medical University, National Center for Children’s Health, Beijing, 100045 China; 2 Department of Child Health Care Center, Beijing Children’s Hospital, Capital Medical University, National Center for Children’s Health, Beijing, 100045 China; 3 Department of Diet and Nutrition, Beijing Children’s Hospital, Capital Medical University, National Center for Children’s Health, Beijing, 100045 China

**Keywords:** Gut microbiota, Antibiotics, Adipokines, Obesity, DNA methylation

## Abstract

**Background:**

Adiponectin and resistin are typically secreted by the adipose tissue and are abnormally expressed in obesity. However, the underlying influential factors and mechanisms are to be elucidated. It is well known that the expression of genes is regulated by epigenetics while gut microbiota participates in epigenetic processes through its metabolites such as folate, biotin, and short-chain fatty acids (SCFAs). Therefore, we supposed that alteration of gut microbiota might affect the transcriptional expression of adiponectin and resistin through epigenetic regulation in obesity.

**Methods:**

C57BL/6J mice were fed either a high-fat diet (34.9% fat by wt., 60% kcal) or a normal-fat diet (4.3% fat by wt., 10% kcal) for 16 weeks, with ampicillin and neomycin delivered via drinking water to interfere with gut microbiota development. Fecal microbiota was analyzed by 16S rRNA high-throughput sequencing. The mRNA expression levels of genes were measured by real-time quantitative RT-PCR. SCFA contents in feces were examined using gas chromatography.

**Results:**

Alteration of the gut microbiota induced by antibiotic use, characterized by a dramatic reduction of the phylum Firmicutes and Actinobacteria and an increase of Proteobacteria with reductions of genera including *Lactobacillus*, *norank_f_Bacteroidales_S24-7_group*, *Alistipes*, *Desulfovibrio*, *Helicobacter*, etc., and increases in *Bacteroides*, *Enterobacter*, *Klebsiella*, inhibited the body weight gain in mice fed the high-fat diet instead of the normal-fat diet. The mRNA expression of adiponectin and resistin was upregulated by antibiotic use in mice fed the high-fat diet, accompanied by increased expression of fat oxidation and thermogenesis-related genes (PPAR-α, Pgc-1α, and Atgl) in the fat and/or liver, whereas no change in the expression of adiponectin and resistin was found in mice fed the normal-fat diet. Furthermore, antibiotic use reduced DNA methylation fractions of the adiponectin and resistin promoters and downregulated the expression of DNA methyltransferase 1 and 3a (DNMT1 and DNMT3a) with the high-fat diet feeding.

**Conclusion:**

Alteration of gut microbiota induced by antibiotic use may affect the expression of adiponectin and resistin in mice fed the high-fat diet by modifying promoter DNA methylation, thus leading to increased fatty acid oxidation and less body weight gain.

## Introduction

Obesity has become one of the most serious global public health challenges of the twenty-first century, but the underlying molecular etiologies and mechanisms still remain enigmatic [1]. Since the ob gene was cloned in 1994 and subsequently its product leptin, primarily expressed in adipocytes, was found to have functions in regulating satiety and energy metabolism [2–4], several other adipokines including adiponectin and resistin have been found and highlighted in obesity pathogenesis and its associated complications [5, 6]. Adiponectin serves as a protective factor to prevent obesity occurrence and/or progression by suppressing inflammation, promoting fatty acid oxidation, and improving insulin sensitivity [7, 8], while resistin is important in maintaining homeostasis of insulin action, energy, glucose, and lipids [5, 6]. Studies from humans and animal models have demonstrated that adiponectin expression is reduced in obesity [7, 8], whereas the expressional change of resistin in obesity is still in controversy owing to the influence of several factors such as types of body fat deposition (central or subcutaneous), genetic background, and gender [9, 10].

In recent years, epigenetic patterns have been found to participate in regulating the expression of genes associated with appetite, energy metabolism, adiposity, etc. [11]. It has been reported that changes in epigenetic modification of leptin and adiponectin genes are associated with occurrence of obesity and other metabolic diseases [12–14]. Therefore, the accumulating evidence shows that the recent rapid increase in obesity rates could be explained by the epigenetic inheritance affected by lifestyle and nutrition in the whole life cycle, which include chemical stressors (metals, air pollution or endocrine-disruptive chemicals such as bisphenol A, etc.), unhealthy habits (tobacco, high alcohol intake, sedentarism, etc.), pharmacological factors, and diet [15].

During the past decade, gut microbiota has been received more attention because of its association with noncommunicable chronic diseases (NCD) including obesity, diabetes, and neurodegenerative diseases. It is clear that gut microbiota, interacting with host genetic and diet, facilitates an important role in regulating energy metabolism and fat storage [16–18]. In obesity, disturbances in the gut microbiota and variations in the ratio between the two major intestinal microbial phyla (Firmicutes and Bacteroidetes) have been reported [16, 17] with conflicting results showing increase, reduction, or no changes in the Firmicutes/Bacteroidetes ratio [16, 19–22]. Besides increasing the host’s ability to harvest energy from the digested food, the disturbed microbiota produces metabolites and microbial products such as short-chain fatty acids (SCFAs) (acetate, propionate, butyrate, etc.), secondary bile acids, and lipopolysaccharides, to modulate appetite, gut motility, energy storage, and expenditure mediated by several pathways including the G-protein-coupled receptor (GPR) targeted genes’ expression and associated metabolism [16–18]. As well, the bioactive substances such as folate, biotin, and SCFAs produced by the intestinal microorganisms participate in epigenetic regulation on the associated genes’ expression, being one-carbon unit donors or potent inhibitors of histone deacetylases (HDACs) [23, 24].

Therefore, we hypothesized that changes in gut microbiota may affect the expression of adiponectin and resistin in obesity, and the underlying mechanisms may be involved in gene epigenetic modification. In this study, using a high-fat diet-induced obese mouse model, we determined the effects of alteration in gut microbiota with antibiotic use on promoter methylation of adiponectin and resistin and their resulted expression.

## Materials and methods

### Diets

A high-fat diet (34.9% fat by wt., 60% kcal) (No. H10060) and a normal-fat diet (4.3% fat by wt., 10% kcal) (No. H10010) were designed based on the formula of diet-induced obesity (D12492) and the control formula (D12450B) from the Research Diets, Inc. (New Brunswick, NJ). The diets were manufactured by Beijing Huafukang Bioscience Co. Inc. (Beijing, China) and stored at − 20 °C until use.

### Animals

Three- to 4-week-old male C57BL/6J mice were purchased from Beijing Huafukang Bioscience Co. Inc. (Beijing, China) and were housed at the animal facilities under a 12 hour (h) light-12 h dark cycle with cycles of air ventilation and temperature and humidity controlled and free access to water and food in the Institute of Laboratory Animal Sciences, Chinese Academy of Medical Sciences and Peking Union Medical College (CAMS&PUMC). After 1 week of recovery from transportation, mice were randomly separated into four groups: diet-induced obesity (DIO), DIO with antibiotics (DIO-AB), normal control (NC), and NC with antibiotics (NC-AB) (*n* = 10 in each group). The DIO and DIO-AB groups were maintained on the high-fat diet and the NC and NC-AB groups were fed the normal-fat diet for 16 weeks. To interfere with gut microbiota, ampicillin (1 g/L) and neomycin (0.5 g/L) (Sigma-Aldrich) targeting mainly gram-positive and gram-negative bacteria respectively were delivered via drinking water as described [25] to mice in the DIO-AB and NC-AB groups during the whole feeding cycle. The body weight was measured weekly for 16 weeks with records of food intake at 8, 12, and 16 weeks after feeding and antibiotic intervention, and the fresh stool samples were collected during the week at 16 after feeding and stored at − 80 °C for later microbiota analysis. At the end of feeding procedure, the 12-h fasted mice were anesthetized by intraperitoneal injection of Avertin (125 mg kg^−1^ of 2,2,2-tribromoethanol, T-4840-2, Sigma-Aldrich Chemie GmbH, Steinheim, Germany) to obtain blood samples by heart puncture, and then euthanized by injection of an overdose of Avertin (500 mg/kg) and decapitation to minimize suffering. After euthanasia, the mouse epididymal fat and liver were dissected free of the surrounding tissue, removed, wrapped in aluminum foil, and frozen in liquid N_2_, and then transferred to − 80 °C for use.

The animal experiments were performed from 08:00 to 12:00 to avoid the negative effects of over fasting on targeted parameters. All experimental protocols (No.2017-01-bch) were approved by the Committee on the Ethics of Institute of Laboratory Animal Sciences, CAMS&PUMC, and in accordance with the Animals (Scientific Procedures) Act, 1986 (UK) (amended 2013), as well as the Guide for the Care and Use of Laboratory Animals of National Administration Regulations on Laboratory Animals of China. All sections of this report adhere to the ARRIVE Guidelines for reporting animal research (www.nc3rs.org.uk).

### Biochemical and metabolic analysis in plasma and feces

Plasma concentrations of triglycerides and cholesterol were assayed by enzymatic procedures, gliseril phospo para amino phenazone (GPO-PAP), and cholesterol oxidase p-aminophenol (COD-CE-PAP) using commercial kits (Sichuan MAKER Science Tech. Co., Ltd., China). SCFAs in feces were examined using gas chromatography (GC) according to our previously described method [26] on an Agilent 6890N GC system equipped with a flame ionization detector (FID) and a high-resolution gas chromatography column of 30 m × 0.25 mm i.d. coated with 1.40 μm film thickness (DB-624UI, J&W Scientific, Agilent Technologies Inc., USA).

### Histological analysis

Three samples from each group were randomly selected for histological analysis. Specifically, about 30 mg of the epididymal fat was fixed in 10% (volume/volume) formaldehyde for 48 h, and then sectioned with CryoStar NX50 cryostat (8 μm). The sections were picked up on a glass slide and stained with hematoxylin-eosin (HE) by professional technicians (Servicebio Science Tech. Co., Ltd., Wuhan, China). Then, stained sections were scanned by a digital slide scanner (Pannoramice MIDI, Hungary), and Case Reviewer software was used to take photograph at appropriate magnification (× 100). Finally, the Image-pro Plus software was used to quantitatively analyze the size of fat cells.

### Analysis of gene mRNA expression

Total RNA was extracted from about 80 mg of the epididymal fat using the Trizol reagent (Invitrogen, Carlsbad, CA, USA), and the cDNA was reversely transcribed using the Quant Script RT Kit (Tiangen Biotech, Beijing, China). The expressional mRNA levels of targeted genes, adipokines (adiponectin and resistin), acetyl-CoA carboxylase (Acc1), adipose triglyceride lipase (Atgl), cell death-inducing DNA fragmentation factor-alpha-like effector A (Cidea), fatty acid synthase (Fas), peroxisome proliferator-activated receptor-alpha (PPAR-α), peroxisome proliferator-activated receptor-γ coactivator 1a (Pgc-1α), and three isoforms DNA methyltransferases (DNMT 1, 3a, and 3b) were measured by the real-time quantitative RT-PCR (CFX-96, Bio-Rad, USA) with β-actin or glyceraldehyde-3-phosphate dehydrogenase (Gapdh) as the invariant internal control. The assays were performed in triplicates, and the results were normalized to the internal standard mRNA levels using the 2^–ΔCT^ method. The oligonucleotide primers for the targeted genes were designed by Primer-Blast (http://www.ncbi.nlm.nih.gov/tools/primer blast/) and shown in the Supplementary Table S1. Furthermore, correlative analysis was conducted between the expression levels of adiponectin and resistin and fecal contents of acetate, propionate, and butyrate.

### Bisulfite conversion and sequencing

Methylation of promoters of adiponectin and resistin genes was analyzed by bisulfite sequencing according to our previous method [15]. The genome DNA was isolated and purified from the epididymal fat using a TIANamp Genomice DNA Kit (Tiangen Biotech, Beijing, China), and then the bisulfite conversion was performed using the Methylamp^TM^ DNA Modification Kit (Cat. No. P-1001, Epigentek Group Inc. Brooklyn, NY). The converted DNA was amplified by nested PCR using Taq DNA Polymerase Master Mix (cat. no. KT201, Tiangen Biotech Inc., Beijing, China) and specific primers for the adiponectin and resistin promoters, which were designed using Methprimer software (Supplementary Table S2). The examined promoter regions of adiponectin and resistin were listed in the Supplementary Figure S1. The adiponectin promoter includes nts 23145384-23146082 and spans 6 CpGs within nts -1162 to -494 (with respect to the TSS), and the resistin promoter region includes nts 3654370-3655769 and spans 18 CpGs within nts -1450 to -113 (with respect to the TSS). The methylation fraction was calculated from the amplitude of cytosine and thymine within each CpG dinucleotide, C/(C+T)*100.

### Gut microbial profiling analysis

About 80 mg feces from each mouse were used to extract total bacterial DNA using a QIAamp DNA Stool Mini Kit (cat. no. 51504, Qiagen, Germany) according to the manufacture’s protocol. The DNA concentration was measured by NanoDrop 2000 spectrophotometer (Thermo Scientific, Wilmington, USA), and DNA purity was evaluated by checking optical density ratio at 260/280 nm as well as 1% agarose gel electrophoresis. Then, the V3-V4 region of bacteria 16S rRNA gene was amplified by PCR and the resulted PCR products were extracted and quantified. Purified amplicons were pooled in equimolar and paired-end sequenced (2 × 300) on an Illumina MiSeq platform (Illumina, San Diego, USA) according to the standard protocols by Majorbio Sanger Bio-Pharm Technology Co. Ltd. (Beijing, China) [26]. Then, raw fastq files were demultiplexed, quality-filtered by Trimmomatic, and merged by FLASH with the following criteria: (i) reads were truncated at any site receiving an average quality score < 20 over a 50 bp sliding window; (ii) primers were exactly matched allowing 2 nucleotide mismatching, and reads containing ambiguous bases were removed; and (iii) sequences whose overlap longer than 10 bp were merged according to their overlap sequence. Operational taxonomic units (OTUs) were clustered with 97% similarity cutoff using UPARSE (version 7.1 http://drive5.com/uparse/). Chimeric sequences were identified and removed using UCHIME. The taxonomy of each 16S rRNA gene sequence was analyzed by RDP Classifier algorithm (http://rdp.cme.msu.edu/) against the Silva (SSU123) 16S rRNA database using confidence threshold of 70%. According to the cluster information, community richness parameters (Ace and Chao) community diversity parameters (Shannon and Simpson) and sequences were calculated using Mothur software. After phylogenetic allocation of the sequences down to the phylum, class, order, family, and genus levels, the relative abundance of a given phylogenetic group was defined as the number of sequences affiliated with that group divided by the total number of sequences per sample. Non-metric multidimensional scaling (NMDS) based on OTU compositions was determined using Qiime software for calculating the beta diversity distance matrix, and the R language vegan software package for NMDS analysis. Linear discriminant analysis (LDA) effect size (LEfSe), which takes into account both statistical significance and biological relevance, was conducted to identify OTUs deferentially represented among the four groups. The non-parametric factorial Kruskal-Wallis (KW) sum-rank test was used to detect the significant difference in abundance, and LDA was used to estimate the effect of abundance of each component on the different effect [26].

### Statistical analysis

Statistical analysis was performed with SPSS software (version 17.0 for Windows). One-way analysis of variance (ANOVA) was performed to compare the means of indexes among different groups with normally distributed data. Mann-Whitney *U* test and Wilcoxon signed-rank test were used for assessing the data with the non-normal distribution, and Dunnett’s T3 test was used for analyzing the data lacking homogeneity of variance. Then, the Student-Newman-Keuls (SNK) test was used to determine where the differences exist between each two groups. Linear relationships between the variables were tested by Spearman’s correlations. *P* < 0.05 was considered to be statistically significant.

## Results

### Antibiotic use inhibited body weight gain in the DIO mice

**Fig. 1:**
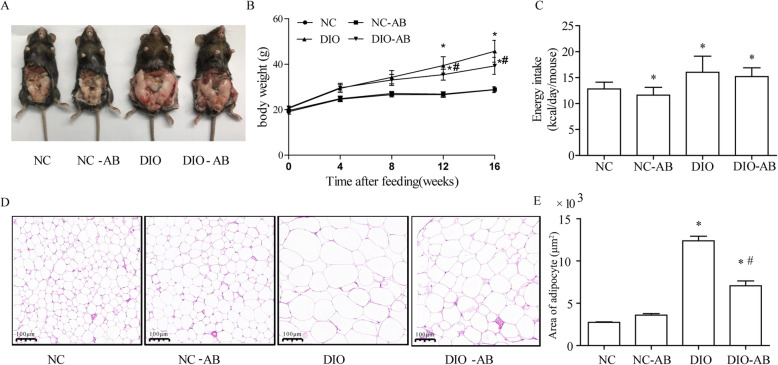
Effects of antibiotics on body weight gain in the high-fat diet-fed mice. Three to 4-week-old C57BL/6J male mice were fed a high-fat diet to induce obesity (DIO group) for 16 weeks, with a normal-fat diet as control (NC group). Meanwhile, ampicillin (1 g/L) and neomycin (0.5 g/L) were delivered via drinking water to mice fed the high-fat diet (DIO-AB group) and the normal-fat diet (NC-AB group). Antibiotic use lessened the weight gain (**a**, **b**) with no changes in energy intake (**c**), and reduced adipocyte size (**d**, **e**). *n* = 10 in each group. Data are means ± SD. *Compared to the NC group, *P* < 0.05; ^#^compared to the DIO group, *P* < 0.05

As shown in Fig. 1a, b, mice from the DIO and DIO-AB groups had a much heavier weight than those from the NC group with a higher food energy intake (*P* < 0.05). With antibiotic use, mice in the DIO-AB group had 14% less body weight gain after 16 weeks of feeding (*P* < 0.05) with no changes in food intake, compared to the DIO group; whereas no differences in body weight change were found between the NC-AB and NC groups, although daily food intake was a little lower in the NC-AB compared to the NC group (Fig. 1c). Histological analysis showed smaller adipocyte size in the DIO-AB group than that in the DIO group (*P* < 0.05), whereas no differences in adipocyte size were shown between the NC-AB and NC groups (Fig. 1d, e).

### Antibiotic use affected plasma lipids

Compared to the DIO group (1.07 ± 0.17 and 3.19 ± 0.55 mmol/L), plasma triglyceride and total cholesterol concentrations in the DIO-AB group (0.75 ± 0.05 and 2.30 ± 0.34 mmol/L) were reduced (*P* < 0.05), resulting in their levels closer to the NC group (0.64 ± 0.10 and 2.04 ± 0.25 mmol/L). This was in keeping with the less weight gain in DIO-AB mice. Plasma triglyceride concentration (0.49 ± 0.11 mmol/L) in the NC-AB group was lower than that in the NC group (*P* < 0.05), with no change in total cholesterol concentration (1.75 ± 0.27 mmol/L).

### Antibiotic use dramatically altered gut microbiota compositions

A dataset of 1,022,080 quality-filtered sequence reads from 40 samples were generated, among which 100% were assigned at the phylum level and 99.98%, 99.98%, 99.88%, and 97.34% at the class, order, family, and genus levels, respectively. Taxonomy-based analysis of the assigned sequences showed that fecal microbial composition of all mice with antibiotics or no antibiotics at the phylum level was comprised of Firmicutes (average 33.00% or 0.76%), Bacteroidetes (51.08% or 43.25%), Proteobacteria (11.63% or 41.12%), Verrucomicrobia (1.64% or 14.86%), Actinobacteria (1.91% or 0.01%), and others (0.77% or 0.01%) (Fig. 3a–e). Both the bacterial richness reflected by the Ace and Chao indexes and the bacterial diversity expressed as Shannon and Simpson were decreased in antibiotic groups compared with the corresponding controls (*P* < 0.05) (Table 1). Non-metric multidimensional scaling (NMDS) analysis indicated that the identified taxa from the DIO-AB and NC-AB groups and the DIO and NC groups were successfully partitioned into two distinct sections respectively at the phylum or genus level (Fig. 3f, g), and the significant differences were found by using the KW test analysis. These data indicate that antibiotic use dramatically changed the profiles of gut microbiota both in the normal-fat or the high-fat diet feeding.

**Table 1:** Characteristics of bacterial sequences in different groups

Sequences					Diversity and richness			
	Valid	Normalization	OTU	Coverage (%)	Ace	Chao	Shannon	Simpson
NC	381,500	300,670	3618	99.77 ± 0.00	404.73 ± 17.71	406.27 ± 19.92	4.09 ± 0.14	0.04 ± 0.01
NC-AB	385,442	300,670	654	99.89 ± 0.00	143.14 ± 37.31*	108.87 ± 22.21*	2.00 ± 0.14*	0.19 ± 0.03*
DIO	372,402	300,670	3314	99.77 ± 0.00	380.34 ± 19.30*	379.55 ± 23.48*	4.26 ± 0.15*	0.03 ± 0.01*
DIO-AB	365,196	300,670	474	99.91 ± 0.00	120.19 ± 48.38*^#^	87.36 ± 30.22*^#^	1.57 ± 0.09*^#^	0.24 ± 0.03*^#^

Three to 4-week-old C57BL/6J male mice were fed a high-fat diet to induce obesity (DIO group) for 16 weeks, with a normal-fat diet as control (NC group). Meanwhile, ampicillin (1 g/L) and neomycin (0.5 g/L) were delivered via drinking water to mice fed the high-fat diet (DIO-AB group) and the normal-fat diet (NC-AB group). Fecal microbiota was analyzed by 16S rRNA high-throughput sequencing. The number of OTUs, coverage percentages, richness estimators (ACE and Chao), and diversity indices (Shannon and Simpson) were calculated at 3% distance. *n* = 10 in each group. Data are means ± SD

*Compared to the NC group, *P* < 0.05

^#^Compared to the DIO group, *P* < 0.05

**Fig. 2:**
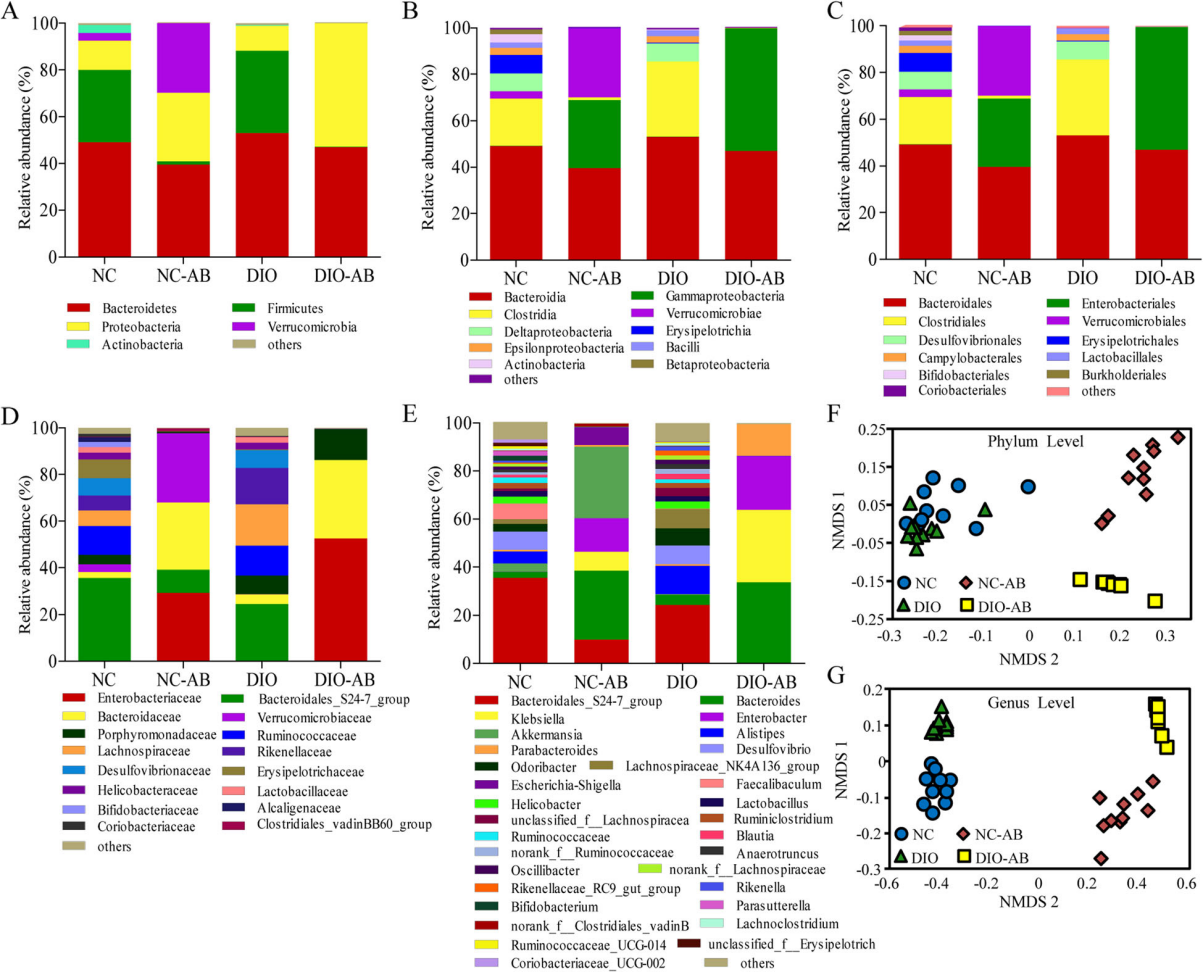
Effects of antibiotics on gut microbiota compositions. The mouse experimental procedure was the same as in Fig. 1. **a**–**e** Community bar-plot analysis on the fecal microbiota compositions at the levels ranged from the phylum, class, order, and family to genus. **f**, **g** Bacterial communities at the phylum and genus levels based on weighted UniFrac distance using the NMDS analysis. *n* = 10 in each group

The patterns seen in microbial compositions were distinctly different between the antibiotic group and the corresponding control group fed either the high-fat diet or the normal-fat diet (Fig. 2a–e, Table 2). Both in the normal-fat diet and the high-fat diet feeding, mice with antibiotics had a greater increase in the proportion of Proteobacteria and a little decrease in Bacteroidetes, with loss of Firmicutes and Actinobacteria at the phylum level, compared to those with no antibiotics (*P* < 0.05). The proportion of Verrucomicrobia in mice with the normal-fat diet feeding was greatly increased after antibiotic use (*P* < 0.05), whereas the reduced Verrucomicrobia in mice with high fat feeding was not affected by antibiotic use. Analysis of bacterial groups at the genus level showed that antibiotic use reduced proportions of most genera including *Faecalibaculum*, *Lactobacillus*, *Ruminococcaceae*, *Blautia*, *Alistipes*, *Odoribacter*, *Bifidobacterium*, *Desulfovibrio*, *Helicobacter*, and *Parasutterella* either with the normal-fat diet or the high-fat diet feeding (*P* < 0.05). However, the proportions of *Bacteroides*, *Enterobacter*, and *Klebsiella* were increased both in the normal-fat and the high-fat feeding (*P* < 0.05) with a greater change in the high-fat feeding, and the *Escherichia-Shigella* and *Akkermansia* were increased only in mice with the normal-fat diet feeding (*P* < 0.05).

**Table 2:** Taxonomic classification of pyrosequences from bacterial communities at the phylum and genus levels

	NC	NC-AB	DIO	DIO-AB
**Firmicutes**	30.87 ± 6.35	1.37 ± 0.81*	35.12 ± 4.21	0.14 ± 0.05*^#^
*Faecalibaculum*^†^	6.45 ± 3.21	0.00 ± 0.00*	0.29 ± 0.17*	0.00 ± 0.00*^#^
*Lactobacillus*^†^	2.44 ± 1.60	0.00 ± 0.00*	2.32 ± 2.53	0.02 ± 0.01*^#^
*unclassified_f__Ruminococcaceae*^†^	2.39 ± 1.40	0.00 ± 0.00*	1.58 ± 0.48	0.01 ± 0.00*^#^
*Ruminiclostridium*^†^	2.25 ± 0.60	0.01 ± 0.01*	1.93 ± 0.60	0.01 ± 0.01*^#^
*Lachnospiraceae_NK4A136_group*	1.89 ± 0.64	0.00 ± 0.01*	7.80 ± 1.22*	0.01 ± 0.01*^#^
*unclassified_f__Erysipelotrichaceae*	1.51 ± 0.90	0.00 ± 0.00*	0.03 ± 0.02*	0.00 ± 0.00*^#^
*norank_f__Clostridiales_vadinBB60_group*	0.20 ± 0.09	1.21 ± 0.83*	0.28 ± 0.19	0.00 ± 0.00*^#^
*Oscillibacter*	1.18 ± 0.42	0.00 ± 0.01*	1.79 ± 0.26	0.00 ± 0.00*^#^
*norank_f__Lachnospiraceae*	1.14 ± 0.28	0.00 ± 0.00*	1.75 ± 0.40	0.01 ± 0.01*^#^
*Anaerotruncus*	1.13 ± 0.43	0.00 ± 0.00*	2.01 ± 0.82*	0.00 ± 0.01*^#^
*norank_f__Ruminococcaceae*^†^	1.11 ± 0.23	0.00 ± 0.00*	2.11 ± 0.58*	0.00 ± 0.00*^#^
*Blautia*	1.07 ± 0.92	0.00 ± 0.00*	2.26 ± 1.65	0.01 ± 0.01*^#^
*Ruminococcaceae_UCG-014*^†^	1.07 ± 0.67	0.00 ± 0.00*	0.51 ± 0.27*	0.00 ± 0.00*^#^
*unclassified_f__Lachnospiraceae*	0.93 ± 0.32	0.01 ± 0.01*	3.42 ± 0.67*	0.01 ± 0.01*^#^
*Lachnoclostridium*	0.64 ± 0.20	0.00 ± 0.00*	1.01 ± 0.22*	0.00 ± 0.00*^#^
**Bacteroidetes**	49.12 ± 6.37	39.54 ± 6.67*	53.03 ± 7.02	46.96 ± 5.67*
*norank_f__Bacteroidales_S24-7_group*	35.62 ± 4.25	9.89 ± 6.39*	24.35 ± 2.72*	0.03 ± 0.02*^#^
*Bacteroides*	2.50 ± 2.16	28.70 ± 10.71*	4.25 ± 1.62*	33.73 ± 10.88*^#^
*Alistipes*	5.03 ± 2.00	0.01 ± 0.01*	11.69 ± 2.88*	0.01 ± 0.01*^#^
*Odoribacter*	3.27 ± 2.06	0.00 ± 0.00*	7.18 ± 1.62*	0.01 ± 0.01*^#^
*Parabacteroides*	0.79 ± 0.35	0.70 ± 0.56	0.81 ± 0.28	13.17 ± 7.14*^#^
*Rikenellaceae_RC9_gut_group*	0.55 ± 0.24	0.00 ± 0.00*	2.14 ± 0.78*	0.00 ± 0.00*^#^
*Rikenella*	0.80 ± 0.41	0.00 ± 0.00*	1.66 ± 0.64*	0.00 ± 0.00*^#^
**Actinobacteria**	3.49 ± 1.78	0.01 ± 0.01*	0.32 ± 0.15*	0.01 ± 0.01*^#^
*Bifidobacterium*^†^	2.12 ± 2.10	0.00 ± 0.00*	0.01 ± 0.01*	0.00 ± 0.00*^#^
*Coriobacteriaceae_UCG-002*	1.26 ± 0.92	0.00 ± 0.00*	0.21 ± 0.14*	0.00 ± 0.00*^#^
**Proteobacteria**	12.54 ± 7.37	29.35 ± 3.70*	10.71 ± 5.55	52.88 ± 5.65*^#^
*Enterobacter*	0.05 ± 0.01	13.92 ± 4.02*	0.04 ± 0.03	22.47 ± 2.84*^#^
*Klebsiella*	0.03 ± 0.02	7.82 ± 2.23*	0.07 ± 0.06	30.00 ± 4.21*^#^
*Escherichia-Shigella*	0.01 ± 0.01	7.57 ± 3.70*	0.02 ± 0.03	0.00 ± 0.00*^#^
*Desulfovibrio*	7.48 ± 3.85	0.00 ± 0.00*	7.68 ± 3.26	0.02 ± 0.02*^#^
*Helicobacter*	2.95 ± 4.87	0.00 ± 0.00*	2.83 ± 2.39	0.01 ± 0.01*^#^
*Parasutterella*	1.96 ± 059	0.00 ± 0.00*	0.01 ± 0.01*	0.00 ± 0.00*
**Verrucomicrobia**	3.23 ± 3.33	29.72 ± 9.29*	0.04 ± 0.03*	0.00 ± 0.00*^#^
*Akkermansia*	3.23 ± 3.33	29.72 ± 9.29*	0.04 ± 0.03*	0.00 ± 0.00*^#^

The mouse experimental procedure was the same as in Table 1. *n* = 10 in each group. Data are means ± SD

^†^SCFA producing bacteria

*Compared to the NC group, *P* < 0.05

^#^Compared to the DIO group, *P* < 0.05

To identify the bacterial taxa with sequences affected by antibiotic use, a metagenomic biomarker discovery approach (LEfSe) was applied to assess the effect size of each differentially abundant taxon, and then LDA coupled with effect size measurements were performed to identify the most differentially abundant taxa and a cladogram was generated. We found that a total of 92 taxa sequences were lost with an enrichment of 21 taxa in the NC-AB group (Supplementary Figure S2), and 91 taxa sequences were lost with an enrichment of 15 taxa in the DIO-AB group (Supplementary Figure S3).

### Effects of alteration in gut microbiota by antibiotics on fecal SCFAs and expression of adipokines

**Fig. 3:**
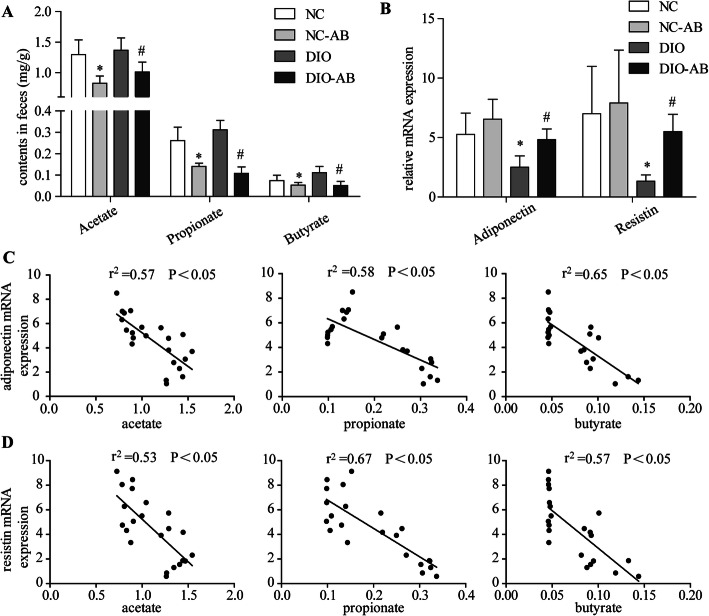
Changes in fecal short-chain fatty acids with antibiotic use and their correlation with the mRNA expression of adipokines. The mouse experimental procedure was the same as in Fig. 1. **a** Contents of acetate, propionate, and butyrate in the feces. **b** Expression of adipose adiponectin and resistin. **c** Correlation between adiponectin and SCFAs. **d** Correlation between resistin and SCFAs. *n* = 5–6 in each group. Data are means ± SD. *Compared to the NC group, *P* < 0.05; ^#^compared to the DIO group, *P* < 0.05

As shown in Fig. 3a, antibiotic use dramatically reduced the contents of acetate, propionate, and butyrate (*P* < 0.05), being independent of the diet type (Fig. 3a). The reduced mRNA expression of adiponectin and resistin in the DIO group, as compared to the NC group, was elevated by antibiotic use (*P* < 0.05), whereas no differences in the two genes’ expression were found between the NC-AB group and the NC group (Fig. 3b). Further analysis indicated that the adiponectin or resistin expression was negatively correlated to the fecal contents of acetate, propionate, and butyrate (*P* < 0.05) (Fig. 3c, d).

### Effects of alteration in gut microbiota by antibiotics on the expression of fatty acid metabolism-associated genes

**Fig. 4:**
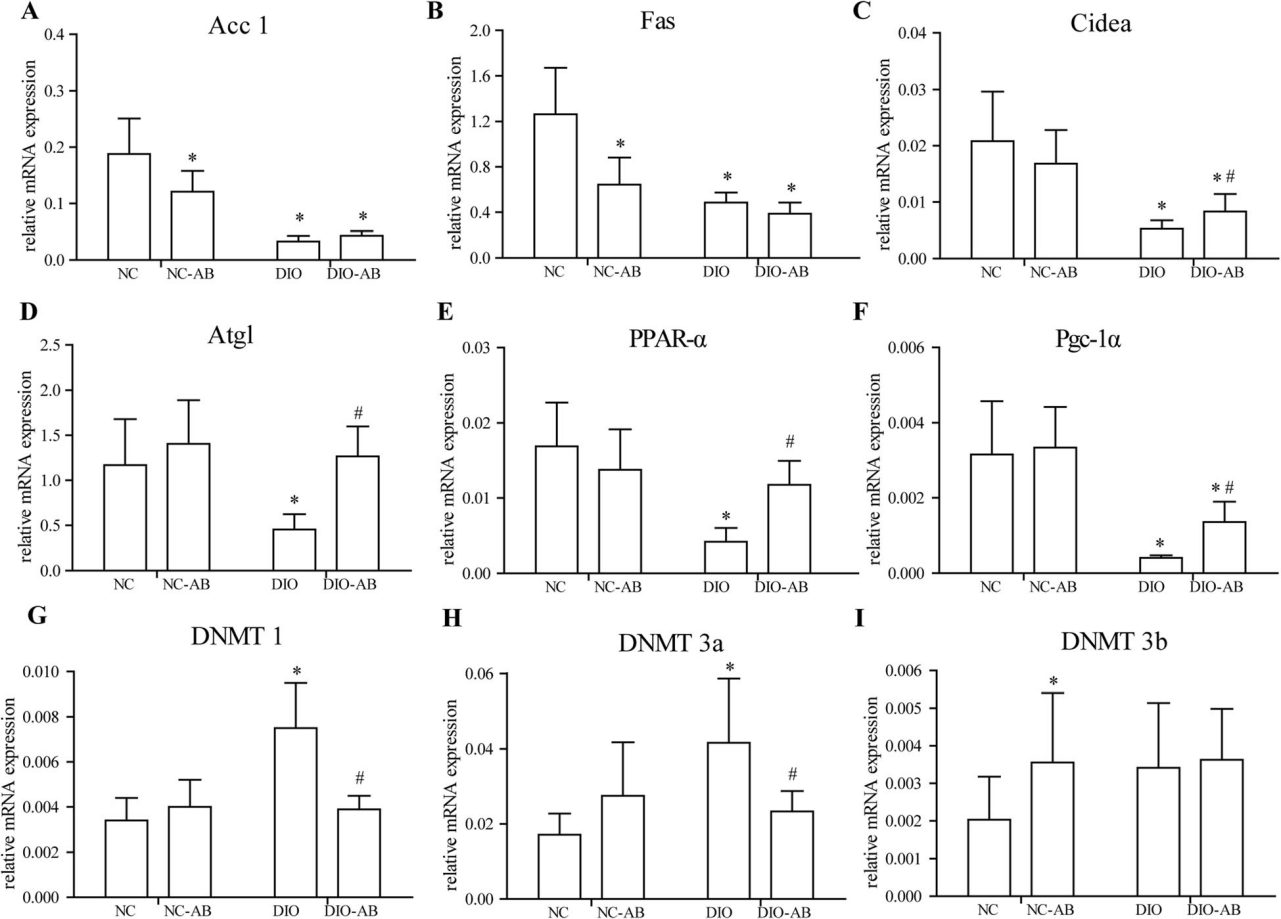
Effects of antibiotics on the mRNA expression of genes associated with fatty acid metabolism and DNA methyltransferase in the adipose tissue. The mouse experimental procedure was the same as in Fig. 1. **a**, **b** Fat synthesis-associated genes, acetyl-CoA carboxylase 1 (Acc1), and fatty acid synthase (Fas). **c**–**f** Fat hydrolysis and oxidation-associated genes, cell death-inducing DNA fragmentation factor-alpha-like effector A (Cidea), adipose triglyceride lipase (Atgl), peroxisome proliferator-activated receptor-alpha (PPAR-α), peroxisome proliferator-activated receptor-γ coactivator1 alpha (Pgc-1α). **g**–**i** DNA methyltransferases 1, 3a, and 3b (DNMT1, DNMT3a, and DNMT3b). *n* = 10 in each group. Data are means ± SD. *Compared to the NC group, *P* < 0.05; ^#^compared to the DIO group, *P* < 0.05

**Fig. 5:**
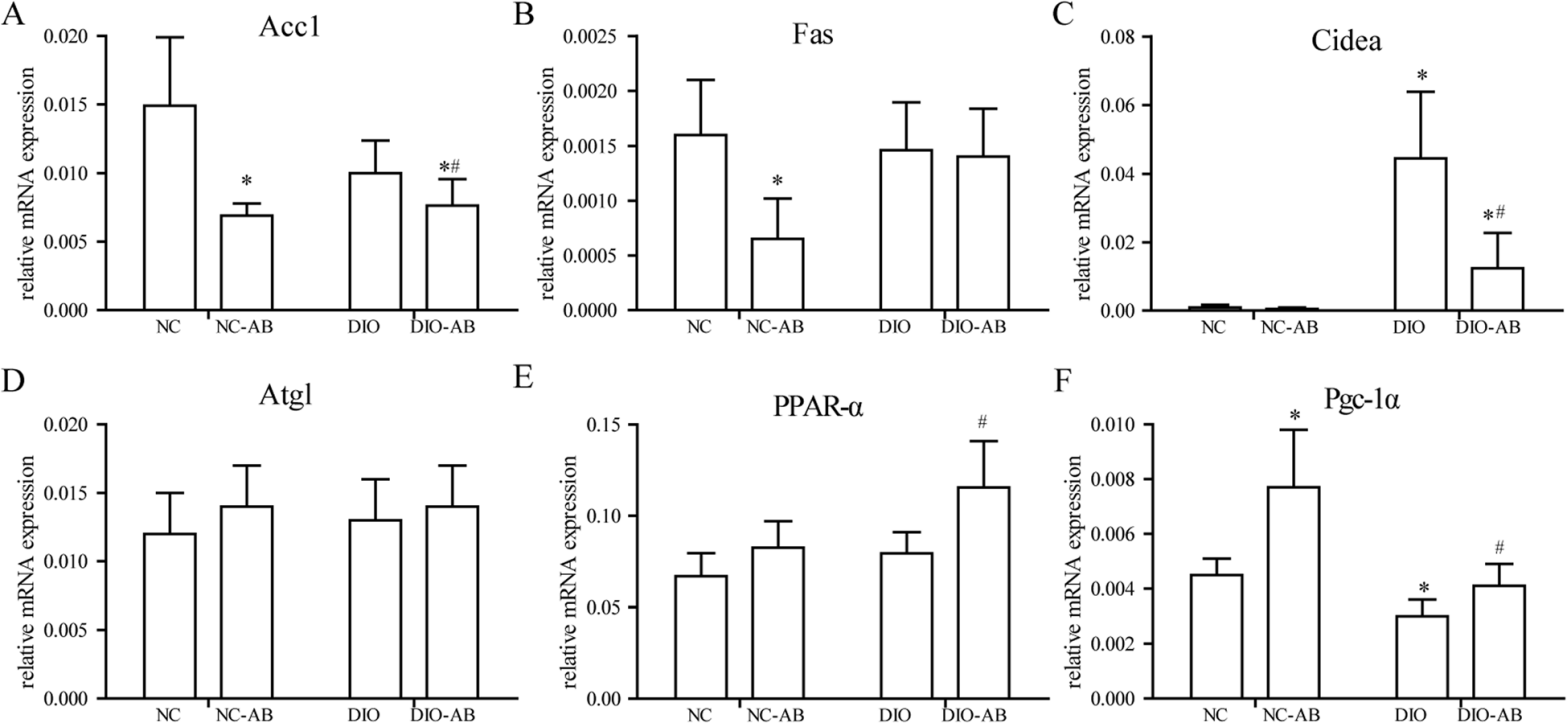
Effects of antibiotics on the mRNA expression of genes associated with fatty acid metabolism in the liver. The mouse experimental procedure was the same as in Fig. 1. **a**, **b** Fat synthesis-associated genes, acetyl-CoA carboxylase 1 (Acc1), and fatty acid synthase (Fas). **c**–**f** Fat hydrolysis and oxidation-associated genes, cell death-inducing DNA fragmentation factor-alpha-like effector A (Cidea), adipose triglyceride lipase (Atgl), peroxisome proliferator-activated receptor-alpha (PPAR-α), peroxisome proliferator-activated receptor-γ coactivator1 alpha (Pgc-1α). *n* = 10 in each group. Data are means ± SD. *Compared to the NC group, *P* < 0.05; ^#^compared to the DIO group, *P* < 0.05

With antibiotic use in the high-fat diet feeding, the expression of fat hydrolysis and oxidation-associated genes (Atgl, PPAR-α, Pgc-1α, and Cidea) in the fat (Fig. 4c–f) and the expression of PPAR-α and Pgc-1α in the liver (Fig. 5e, f) were increased, being with reduced expression of Acc1 and Cidea in the liver (Fig. 5a, c), as compared to no antibiotic use (*P* < 0.05), whereas with antibiotic use in the normal-fat diet feeding, the reduced expression of Acc1 and Fas both in the fat (Fig. 4a, b) and liver (Fig. 5a, b) was found with an increased expression of Pgc-1α in the liver (Fig. 5f), compared to no antibiotic use (*P* < 0.05).

### Effects of alteration in gut microbiota by antibiotics on DNA methylation of the adipokine promoters

In the DIO mice, a total of 5 and 8 CpG sites in the promoters of adiponectin and resistin were hypermethylated compared to the NC mice (*P* < 0.05). Analysis of antibiotic use showed that the averaged fractions of DNA methylation in the promoters of adiponectin and resistin in the DIO-AB mice had a declining trend compared with the DIO mice. Targeting on specific CpG sites, the methylated fractions in 4 CpG sites (sites 2, 4, 5, 6) at the adiponectin promoter and 4 CpG sites (sites 5, 7, 9, 10) at the resistin promoter were reduced by approximately 5% to 10% in the DIO-AB mice compared to the DIO mice (*P* < 0.05). Antibiotic use did not affect DNA methylation in the promoters of adiponectin and resistin in mice fed the normal-fat diet (Table 3). The mRNA detection showed that, with antibiotic use, the expression of DNMT1 and DNMT3a in the fat was downregulated in mice with the high-fat diet feeding (Fig. 4g, h), and the DNMT4b was upregulated in mice with the normal-fat diet feeding (Fig. 4i) (*P* < 0.05).

**Table 3:** Quantitative methylation analysis of the adipokine promoters

Adiponectin	CG2	CG3	CG4	CG5	CG6	Average									
NC	79.6 ± 2.7	72.4 ± 4.4	82.8 ± 1.0	77.6 ± 4.1	69.8 ± 2.4	76.4 ± 5.3									
NC-AB	81.2 ± 4.3	75.4 ± 4.8	83.4 ± 2.2	76.7 ± 1.1	67.0 ± 5.7	76.7 ± 6.3									
DIO	83.9 ± 4.4*	80.4 ± 2.9*	90.7 ± 2.8*	84.0 ± 1.5*	75.9 ± 2.8*	83.0 ± 5.4									
DIO-AB	79.5 ± 4.3^#^	78.2 ± 3.2*	86.2 ± 3.9*^#^	79.5 ± 4.3^#^	69.0 ± 2.6^#^	78.5 ± 6.2									
**Resistin**	**CG2**	**CG3**	**CG5**	**CG6**	**CG7**	**CG8**	**CG9**	**CG10**	**CG11**	**CG12**	**CG13**	**CG14**	**CG15**	**CG16**	**Average**
NC	79.3 ± 7.3	90.7 ± 3.3	79.0 ± 4.8	86.9 ± 3.4	68.2 ± 4.6	80.2 ± 2.9	72.6 ± 4.5	74.3 ± 4.0	65.5 ± 4.2	56.5 ± 2.0	73.3 ± 6.8	78.4 ± 8.7	74.8 ± 4.7	76.2 ± 9.0	75.4 ± 8.5
NC-AB	81.6 ± 5.9	90.2 ± 4.6	81.6 ± 3.9	85.3 ± 3.4	71.1 ± 5.1	76.7 ± 4.0	76.8 ± 5.3	77.5 ± 2.1	67.7 ± 3.8	56.4 ± 2.0	80.4 ± 7.8	80.4 ± 13.4	77.3 ± 5.2	80.7 ± 8.5	77.4 ± 8.2
DIO	91.1 ± 3.8*	93.4 ± 4.9	87.8 ± 5.4*	86.8 ± 5.5	75.7 ± 6.2*	80.9 ± 7.5	79.0 ± 2.0*	82.9 ± 5.5*	69.5 ± 6.6	55.9 ± 1.3	85.1 ± 6.4*	88.8 ± 6.3*	83.5 ± 8.4*	78.5 ± 6.0	81.4 ± 9.7
DIO-AB	84.6 ± 6.9	91.3 ± 4.6	80.4 ± 6.9^#^	85.9 ± 1.2	68.0 ± 5.2^#^	79.5 ± 6.5	75.0 ± 2.8^#^	74.5 ± 5.6^#^	72.3 ± 6.1	58.4 ± 3.1	79.7 ± 7.5	87.3 ± 5.9	75.7 ± 10.2	74.1 ± 4.9	77.6 ± 8.5

Genomic DNA isolated from epididymal fat was analyzed for the methylation of CpG sites at the indicated positions in the promoters of adiponectin and resistin genes spanning nucleotides -1162 to -494 and -1450 to -113 respectively, relative to the TSS. The methylation fraction was calculated from the amplitude of cytosine and thymine within each CpG dinucleotide, C/(C+T)*100. The result for each CpG site is represented as the means ± SD. *n* = 10 in each group

*Compared to the NC group, *P* < 0.05

^#^Compared to the DIO group, *P* < 0.05

## Discussion

The expression of adipokines including leptin, adiponectin, and resistin is dysregulated in obesity and closely associated with secondary metabolic diseases [27]. However, the underlying mechanisms are unclear. In this study, we demonstrated that modification of gut microbiota by ampicillin and neomycin intervention, characterized by reduced richness and diversity with altered proportions of the phyla and genera and elevated adipose expression of adiponectin and resistin with DNA hypomethylation in their promoters, produced inhibitive effects on body weight gain in the high-fat diet-induced obese mice.

Emerging studies have reported that obesity is closely associated with disturbances in gut microbiota with a loss of intestinal microbial diversity and compositional changes *characterized by* a decrease in proportions of the *Bacteroidetes* together with an increase of the *Firmicutes* [19, 27, 28]. In the present study, no change in proportions of Firmicutes and *Bacteroidetes* was shown in the DIO mice, with a reduction of Actinobacteria and Verrucomicrobia. The conflicting results exist owing to many variables involved, such as diet type, genetic background, sample handing, sequencing techniques, and data analysis tools, among others [17]. To address the causality between gut microbiota and diseases, antibiotic treatment and germ-free mice have been conducted [25, 29–32]. It has been demonstrated that antibiotic treatment modifies changes in bile acid and inflammatory signaling, insulin resistance, and glucose and fatty acid metabolism driven by a high-fat diet in mice [29, 30], and these effects are not caused by antibiotic’s direct effects but rather by derived changes in the gut microbiota [33]. However, inconsistent results are reported that interfering with a resilient adult microbiota by antibiotics had no clinically relevant short-term and long-term effects on several metabolic parameters [34], whereas antibiotic use in childhood altered the gut microbiota which was integrally involved in the long-term metabolic programming, predisposing to overweight and obesity [35]. The inconsistency in correlation between antibiotic treatment and weight gain and associated metabolism from both human and animal studies may be due to differences in antibiotic dose, class, period of exposure, and ways of administration [31].

In keeping with Suarez-Zamorano’s report that microbiota depletion stimulates beige fat development and reduces obesity [30], our study demonstrated that antibiotic treatment to mice during the whole feeding process had a suppression of body weight gain in mice with the high-fat diet feeding. Concomitantly, the expression of adiponectin and resistin and genes associated with beta oxidation and thermogenesis (PPAR-α, Pgc-1α, and Atgl) was upregulated, and the expression of fat synthesis associated genes (Acc1 and Fas) was downregulated in the adipose tissue and/or liver. Changes in the expression of genes associated with fat metabolism might be resulted from increased expression of adiponectin in the fat, which upon binding to its receptors initiates a series of tissue microenvironment-dependent signal transduction events, including phosphorylation of adenosine monophosphate (AMPK) and increased PPARα ligand activity, and further stimulates fatty acid oxidation in skeletal muscle [36]. Whether the expression of these genes was involved in the resistin expression needs further investigation because the resistin expression in obesity has been in controversy, and in vitro treatment with resistin leads to a reduction in the rate of cellular fatty acid oxidation [37, 38]. In addition, we found that the expression of Cidea in the liver was upregulated by the high-fat diet, and antibiotic use reduced its expression, and this might result in less hepatic lipid droplet formation and storage because the highly expressed Cidea in the liver of mice is associated with hepatic steatosis under the high-fat diet feeding [39, 40].

Although each class of antibiotics induces different alterations to the gut microbiota compositions, a reduction of phylum Firmicutes and an increase of Proteobacteria with *Bacteroides* were reported by most studies together with reduction of genera *Lactobacillus* and *Bifidobacterium* [29–31]. Similarly, in our study, with mice fed the high-fat diet, the use of ampicillin and neomycin dramatically reduced proportions of the Firmicutes and Actinobacteria with an increase of Proteobacteria and genera *Bacteroides*, leading to a reduction of body weight with a higher beta oxidation and thermogenesis. Consistently, in Fujisaka’s study, treatment by vancomycin or metronidazole abolished the proportion of Bacteroidetes with a reduction of Firmicutes and an increase of Proteobacteria, which improved glucose metabolism and insulin signaling in B6J mice [29]. Suárez-Zamorano et al. reported that similar effects in improving the tolerance to glucose, sensitivity to insulin, increased browning, and thermogenic capacity were produced by a total of nine antibiotics including neomycin, streptomycin, penicillin, vancomycin, metronidazole, bacitracin, ciprofloxacin, ceftazidime, and gentamycin administrated to the obese mice [30]. Interestingly, in this study with the high-fat diet feeding, antibiotic-induced alterations in the phylum Proteobacteria and the subordinated genera *Parabacteroides*, *Enterobacter*, *Klebsiella*, *Escherichia-Shigella*, and *Akkermansia*, being different from those with the normal-fat diet feeding, might be responsible for changes in the expression of adipokines and associated genes and the less weight gain.

The disturbed adipokine expression in obesity is primarily attributable to a failure of transcriptional regulation, which may be influenced by multiple factors including diets, adipocyte hypertrophy, inflammation, and oxidative stress [11, 41]. In recent years, it has been verified that epigenetic changes including DNA methylation are the key players in governing gene expression independently of modulating expression of transcription factors [42, 43] and play important roles in the transcriptional dysregulation of obesity associated genes’ expression [11, 41]. The results presented here suggested that the reduced mRNA expression of adiponectin and resistin might be the result of DNA hypermethylation in their promoters and increased expression of DNMT1 in the DIO mice. This is consistent with Kim’s report that DNA hypermethylation of a particular region of the adiponectin promoter suppressed adiponectin expression through epigenetic control mediated by a higher DNMT1 expression and, in turn, exacerbated metabolic complications in obesity [12]. However, the expression of resistin and its epigenetic modification are still in controversy [34–46]. Kim et al. found that, in the DIO rats, the expression levels of different genes in adipose tissue including resistin were upregulated [45], whereas Nowacka-Woszuk reported reduction in the resistin expression in the DIO rats, and no correlation of DNA methylation with transcript levels were observed [46].

The underlying mechanisms by which antibiotic use modified the mRNA expression of adiponectin and resistin might be involved in the altered gut microbiota, which produces low molecular weight substances such as folate, biotin, and SCFAs that potentially modulate signaling pathways and regulate gene expression by epigenetic modifications including DNA methylation, histone modification, and non-coding RNAs [23, 24, 47, 48]. SCFAs are considered to protect against diet-induced obesity by anti-inflammatory potential and inhibition of adipose tissue expansion, with butyrate and propionate being more efficient than acetate [49, 50]. It has been found that fecal SCFAs and plasma acetate contents in the high-fat diet-fed mice significantly decreased compared with those in the normal-fat diet-fed mice, presumably due to the replacement of carbohydrate with fat in the high-fat diet, which reduced SCFA production by gut microbiota [51]. In contrast, a higher SCFA content in feces of obese humans was found and a higher concentration of butyrate and acetate was measured in the caeca of obese mice [52, 53]. Nonetheless, previously, we have demonstrated that the lower mRNA levels of adiponectin and resistin in obese mice can be reversed to normal range by dietary supplementation of SCFAs, and these effects may be involved in epigenetic modifications through directly reducing the expression of DNMT1, DNMT 3a, and DNMT3b and suppressing the binding of these enzymes to the promoters of adiponectin and resistin [54]. In the present study, with antibiotic use, fecal SCFA contents were reduced due to the reduction of SCFA-producing bacteria including the phylum Firmicutes and the genera of *Faecalibaculum*, *Ruminococcaceae*, *Lactobacillus*, and *Bifidobacterium* [55, 56], which was not concomitant with the reduced methylation fractions of CpG sites at the promoters of adiponectin and resistin and the downregulated DNMT1 expression in the high-fat diet feeding. To note, other bioactive compounds may contribute to these adipokines’ DNA methylation. The lessened proportions of *Bifidobacterium* and *Lactobacillus* after antibiotic use in this study might result in less production of B vitamins including folate [23, 48], thus probably leading to hypomethylation of genome DNA including the adiponectin and resistin genes.

## Conclusion

Alteration of gut microbiota by antibiotic use had beneficial effects on the expression of adiponectin and resistin in the high-fat diet-induced obese mice through modifying DNA methylation of their promoters. These changes in adipokines might promote the beta oxidation and thermogenesis and inhibit fat synthesis by regulating related genes’ expression, thus leading to the less body weight gain with the high-fat diet feeding.

## Supplementary information

**Supplementary Table S1.** Sequences of primers used for RT-PCR.

**Supplementary Table S2.** Bisulfite sequencing primers and annealing temperature in this study

**Supplementary Figure S1.** Regions of the mouse adiponectin and resistin promoters. The CG dinucleotides, assigned to each of the analyzed CGs, were marked and numbered on the top right. (A) The adiponectin promoter sequence with two regions spanning nucleotides -1162 to -455. (B) The resistin promoter sequence with three regions spanning nucleotides -1450 to -113.

**Supplementary Figure S2.** Comparison of relative abundance through cladogram analysis and LDA score between mice in the NC and NC-AB group. LEfSe identifies the most differentially abundant taxa between mice of the NC and NC-AB groups at the level from phylum to genus. A: Cladogram representations of data are shown in panels. The size of each dot is proportional to its effect size. Only taxa meeting an LDA significant threshold of >3 are shown. The decreased abundance of 92 taxa and the increase abundance of a total of 21 taxa could be used to discriminate effects of antibiotic use in mice with normal fat feeding. The size of each dot is proportional to its effect size. B: The NC group enriched taxa are respectively indicated with a positive LDA score (red), and taxa enriched in the NC-AB group have a negative score (blue). Red color, the NC group-enriched taxa; blue color, the NC-AB group-enriched taxa.

**Supplementary Figure S3.** Comparison of relative abundance through cladogram analysis and LDA score between mice in the DIO and DIO-AB group. LEfSe identifies the most differentially abundant taxa between mice of the DIO and DIO-AB groups at the level from phylum to genus. A: Cladogram representations of data are shown in panels. The size of each dot is proportional to its effect size. Only taxa meeting an LDA significant threshold of >3 are shown. The decreased abundance of 91 taxa and the increase abundance of a total of 15 taxa could be used to discriminate effects of antibiotic use in mice with high fat feeding. The size of each dot is proportional to its effect size. B: The DIO group enriched taxa are respectively indicated with a positive LDA score (red), and taxa enriched in the DIO-AB group have a negative score (blue). Red color, the DIO group-enriched taxa; blue color, the DIO-AB group-enriched taxa.

## Data Availability

All data generated or analyzed during this study are included in this published article and its supplementary information files.

## Abbreviations

Acc1: Acetyl-CoA carboxylase 1
Atgl: Adipose triglyceride lipase
DIO: Diet-induced obesity
DNMT: DNA methyltransferases
Fas: Fatty acid synthase
GPRs: G protein-coupled receptors
HDACs: Histone deacetylases
LPS: Lipopolysaccharides
NCD: Noncommunicable chronic diseases
OTUs: Operational taxonomic units
Pgc-1α: Peroxisome proliferator-activated receptor-γ coactivator1 alpha
PPAR-α: Peroxisome proliferator-activated receptor-alpha
SCFAs: Short-chain fatty acids
