# Supplementary material for: Alteration of gut microbiota affects expression of adiponectin and resistin through modifying DNA methylation in high-fat diet-induced obese mice
Source: Genes Nutr. 2020 Jun 26;15:12. doi: 10.1186/s12263-020-00671-3 (PMC7318443; doi:10.1186/s12263-020-00671-3)
Supplement: Supplementary file 1 — Supplementary Table S1. Sequences of primers used for RT-PCR. [file 12263_2020_671_MOESM1_ESM.docx]

| **primer** |  | **sequence** |
| --- | --- | --- |
| Acc1 | forward | GGGCACAGACCGTGGTAGTT |
|  | reverse | CAGGATCAGCTGGGATACTGAGT |
| Fas | forward | GGAGGTGGTGATAGCCGGTAT |
|  | reverse | TGGGTAATCCATAGAGCCCAG |
| Cidea | forward | TCCTCGGCTGTCTCAATG |
|  | reverse | TGGCTGCTCTTCTGTATCG |
| Pgc-1α | forward | AGCCGTGACCACTGACAACGAG |
|  | reverse | GCTGCATGGTTCTGAGTGCTAAG |
| PPAR-α | forward | ACGGCAATGGCTTTATCA |
|  | reverse | CGCTGCGTCGGACTCGGT |
| Adiponectin | forward | TGACGACACCAAAAGGGCTC |
|  | reverse | ACCTGCACAAGTTCCCTTGG |
| Resistin | forward | CCTGCTAAGTCCTCTGCCAC |
|  | reverse | GGCTTCATCGATGGGACACA |
| Atgl | forward | TGACTCGAGTTTCGGATGGAGA |
|  | reverse | GAAATGCCGCCATCCACATAG |
| DNMT1 | forward | AAAGTGTGATCCCGAAGATCAAC |
|  | reverse | TGGTACTTCAGGTTAGGGTCGTCTA |
| DNMT3a | forward | CCGCCTCTTCTTTGAGTTCTAC |
|  | reverse | AGATGTCCCTCTTGTCACTAACG |
| DNMT3b | forward | CCCAAGTTGTACCCAGCAATTC |
|  | reverse | TGCAATTCCATCAAACAGAGACA |
| β-actin | forward | GGCCAACCGTGAAAAGATGA |
|  | reverse | CAGCCTGGATGGCTACGTACA |
| GAPDH | forward | CAATAATGGGGAGAGGTTCG |
|  | reverse | TGCTGCTTCCCGAGTAAAAT |

**Table S1. Sequences of primers used for RT-PCR**
