# Supplementary material for: Alteration of gut microbiota affects expression of adiponectin and resistin through modifying DNA methylation in high-fat diet-induced obese mice
Source: Genes Nutr. 2020 Jun 26;15:12. doi: 10.1186/s12263-020-00671-3 (PMC7318443; doi:10.1186/s12263-020-00671-3)
Supplement: Supplementary file 2 — Supplementary Table S2. Bisulfite sequencing primers and annealing temperature in this study [file 12263_2020_671_MOESM2_ESM.docx]

**Table S2. Bisulfite sequencing primers and annealing temperature in this study**

| **Gene** | **Region** | **Primer sequence** | **Product** | **Annealing** |
| --- | --- | --- | --- | --- |
|  |  |  | **Size (bp)** | **temperature (℃)** |
| Adiponectin | A1 | F: GTGTTTTGTGATATTGGGTTTG | 298 | 55 |
|  |  | Inner R: ACTAACTATTCTTCCAAAAATCCTA |  |  |
|  |  | Outer R: TAAAAACACTAACTATTCTTCCAAA |  |  |
|  | A2 | Outer F: TGGAGGAAGTAGATGTTTGGTTAGT | 179 | 57 |
|  |  | Inner F: TGGTTAGTTTTTGTTTGGGAGTAGT |  |  |
|  |  | R: CAAAACAATACCTTAAAAACCTCTC |  |  |
| Resistin | R1 | Outer F: TTTAGTGAGATGTTTTGGGTAA | 399 | 55 |
|  |  | Inner F: GTTTTTGTTTAGTTTTATTTTTATT |  |  |
|  |  | R: CTACTCAACCCTTCCTACATTA |  |  |
|  | R2 | F: TTTTGTTTTATTTAGTTAAAAGATA | 284 | 52 |
|  |  | Inner R: ATAAATCTTAAATAATCATAACTCC |  |  |
|  |  | Outer R: CATTACCCAAAACATCTCACTA |  |  |
|  | R3 | Outer F: TTAGTAATGTTTGGGAATGGAG | 359 | 54 |
|  |  | Inner F: AGTAATGTTTGGGAATGGAGAT |  |  |
|  |  | R: TTATTCATTAAATCCAAACTCTTTT |  |  |
