# Supplementary material for: Alteration of gut microbiota affects expression of adiponectin and resistin through modifying DNA methylation in high-fat diet-induced obese mice
Source: Genes Nutr. 2020 Jun 26;15:12. doi: 10.1186/s12263-020-00671-3 (PMC7318443; doi:10.1186/s12263-020-00671-3)
Supplement: Supplementary file 3 — Supplementary Figure S1. Regions of the mouse adiponectin and resistin promoters. The CG dinucleotides, assigned to each of the analyzed CGs, were marked and numbered on the top right. (A) The adiponectin promoter sequence with two regions spanning nucleotides -1162 to -455. (B) The resistin promoter sequence with three regions spanning nucleotides -1450 to -113. [file 12263_2020_671_MOESM3_ESM.docx]

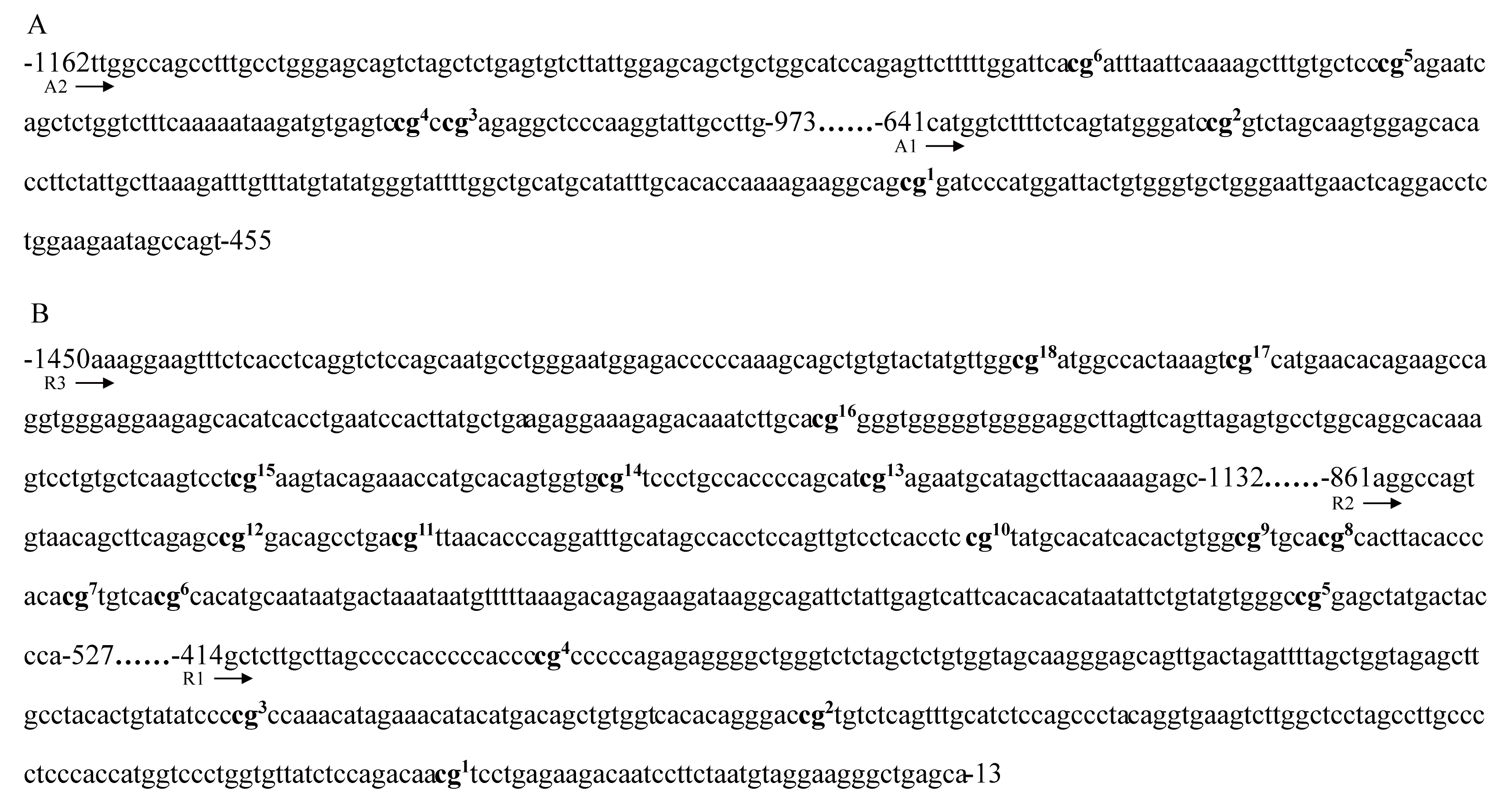
**Figure S1. Regions of the mouse adiponectin and resistin promoters**. The CG dinucleotides, assigned to each of the analyzed CGs, were marked and numbered on the top right. (A) The adiponectin promoter sequence with two regions spanning nucleotides -1162 to -455. (B) The resistin promoter sequence with three regions spanning nucleotides -1450 to -113
