# Supplementary material for: Alteration of gut microbiota affects expression of adiponectin and resistin through modifying DNA methylation in high-fat diet-induced obese mice
Source: Genes Nutr. 2020 Jun 26;15:12. doi: 10.1186/s12263-020-00671-3 (PMC7318443; doi:10.1186/s12263-020-00671-3)
Supplement: Supplementary file 4 — Supplementary Figure S2. Comparison of relative abundance through cladogram analysis and LDA score between mice in the NC and NC-AB group. LEfSe identifies the most differentially abundant taxa between mice of the NC and NC-AB groups at the level from phylum to genus. A: Cladogram representations of data are shown in panels. The size of each dot is proportional to its effect size. Only taxa meeting an LDA significant threshold of >3 are shown. The decreased abundance of 92 taxa and the increase abundance of a total of 21 taxa could be used to discriminate effects of antibiotic use in mice with normal fat feeding. The size of each dot is proportional to its effect size. B: The NC group enriched taxa are respectively indicated with a positive LDA score (red), and taxa enriched in the NC-AB group have a negative score (blue). Red color, the NC group-enriched taxa; blue color, the NC-AB group-enriched taxa. [file 12263_2020_671_MOESM4_ESM.docx]

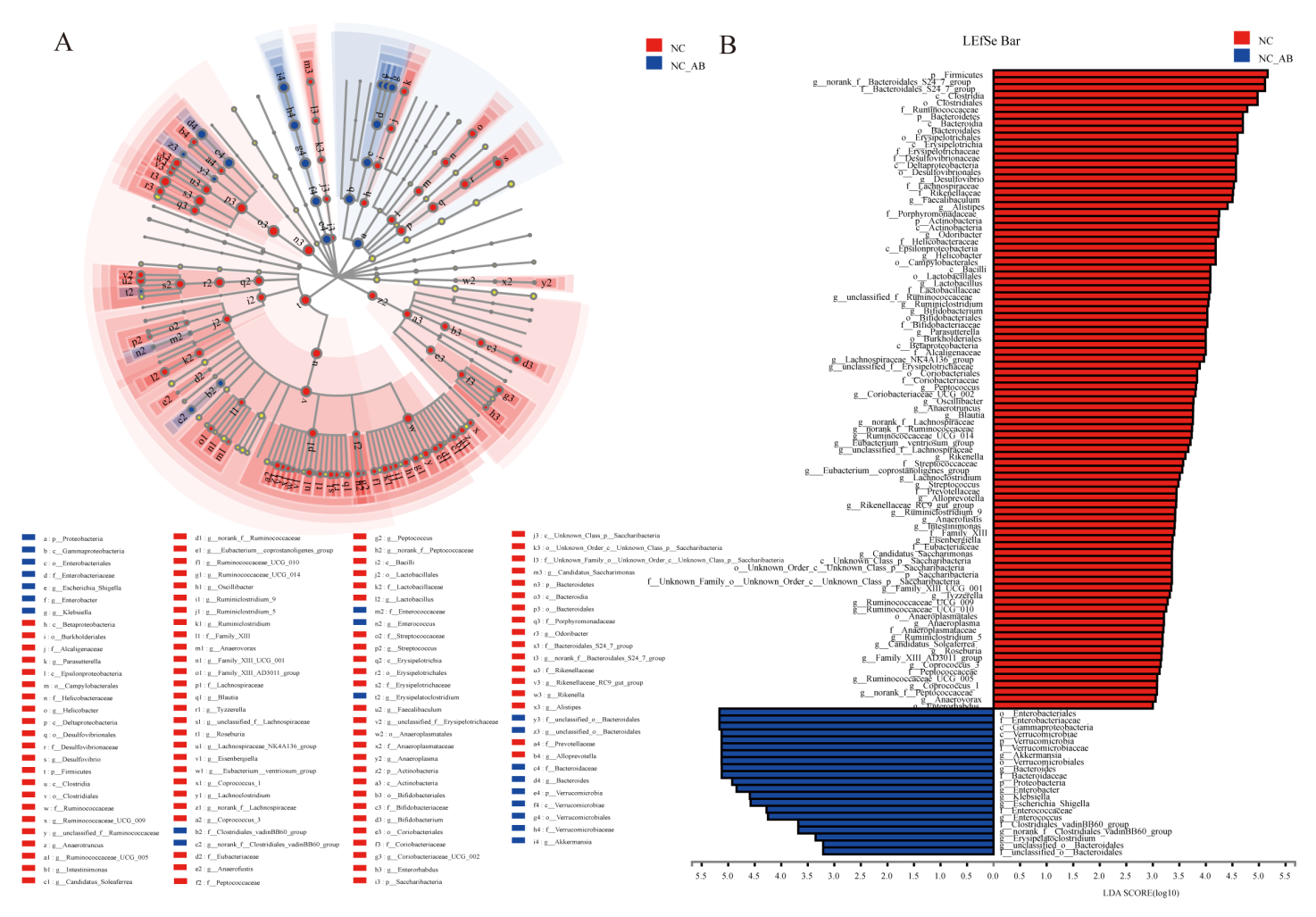
**Figure S2. Comparison of relative abundance through cladogram analysis and LDA score between mice in the NC and NC-AB group.** LEfSe identifies the most differentially abundant taxa between mice of the NC and NC-AB groups at the level from phylum to genus. A: Cladogram representations of data are shown in panels. The size of each dot is proportional to its effect size. Only taxa meeting an LDA significant threshold of >3 are shown. The decreased abundance of 92 taxa and the increase abundance of a total of 21 taxa could be used to discriminate effects of antibiotic use in mice with normal fat feeding. The size of each dot is proportional to its effect size. B: The NC group enriched taxa are respectively indicated with a positive LDA score (red), and taxa enriched in the NC-AB group have a negative score (blue). Red color, the NC group-enriched taxa; blue color, the NC-AB group-enriched taxa
